# Supplementary material for: Microbial diversification is maintained in an experimentally evolved synthetic community
Source: mSystems. 2024 Oct 15;9(11):e01053-24. doi: 10.1128/msystems.01053-24 (PMC11575400; doi:10.1128/msystems.01053-24)
Supplement: Supplemental tables and figures — Fig. S1–S5 and Tables S1 and S2. [file msystems.01053-24-s0001.pdf]

Supplemental materials

Supplemental Figures

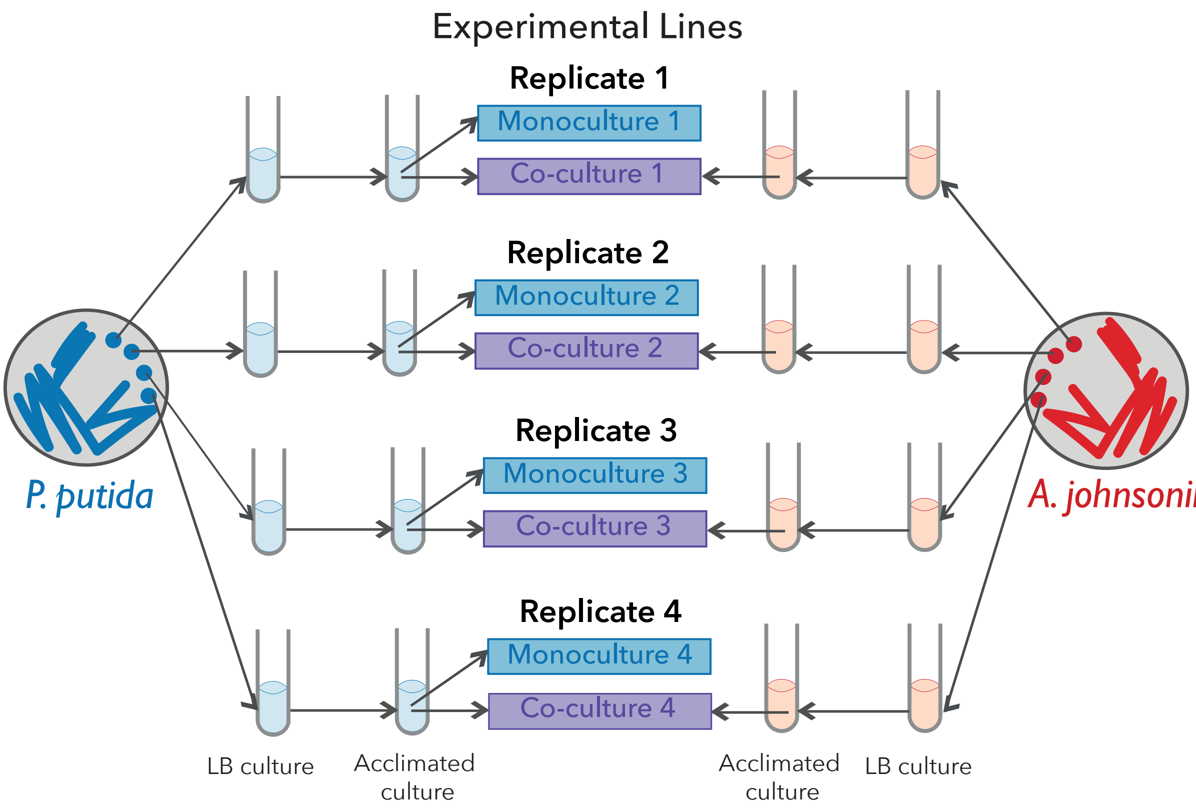

Fig. S1. Design of the evolution experiment. The evolution experiment started with four independent clones (i.e., four ancestors) grown in LB, washed, and acclimated in FAB medium supplemented with 0.6 mM benzoate or 0.6 mM benzyl alcohol to grow *P. putida* and A. *johnsonii*, respectively. We then mixed the acclimated cultures from each species at a ratio of 1:1 for co-cultures or evolved *P. putida* in monoculture.

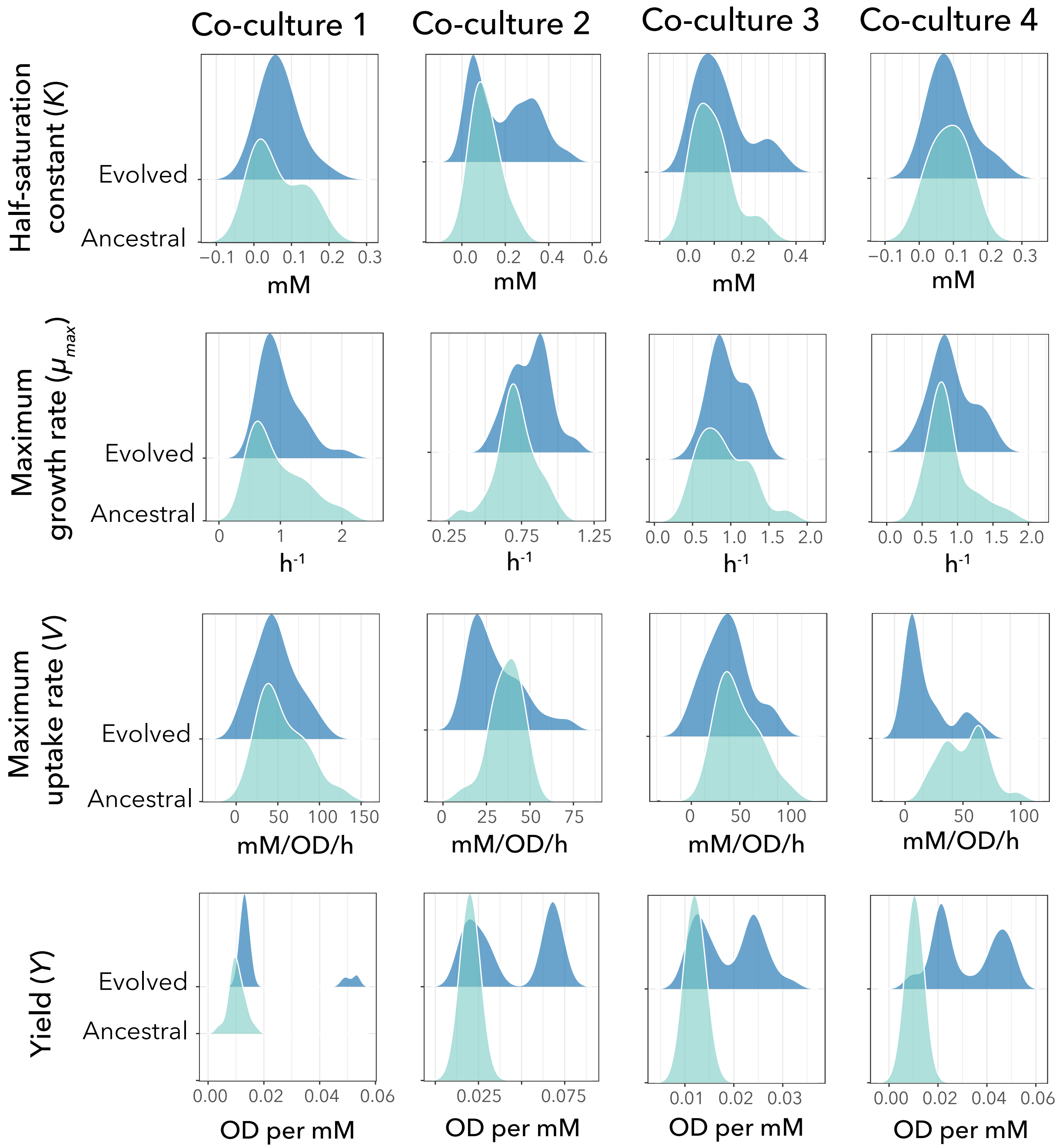

Fig. S2. Distributions of half-saturation constant, maximum growth rate, maximum uptake rate, and yield of the ancestral and evolved *P. putida* in co-culture. Hartigans' dip test for multimodality indicated significant bimodality in the yield of *P putida* evolved in co-culture with *p*-values equal to 0.041, 0.004, $7.48*10^{-6}$, and $2.2*10^{-16}$ for replicates 1, 2, 3, and 4 respectively.

The distribution data were plotted using the `geom_density_ridges` from the `ggridges` package in R, which smooths the data.

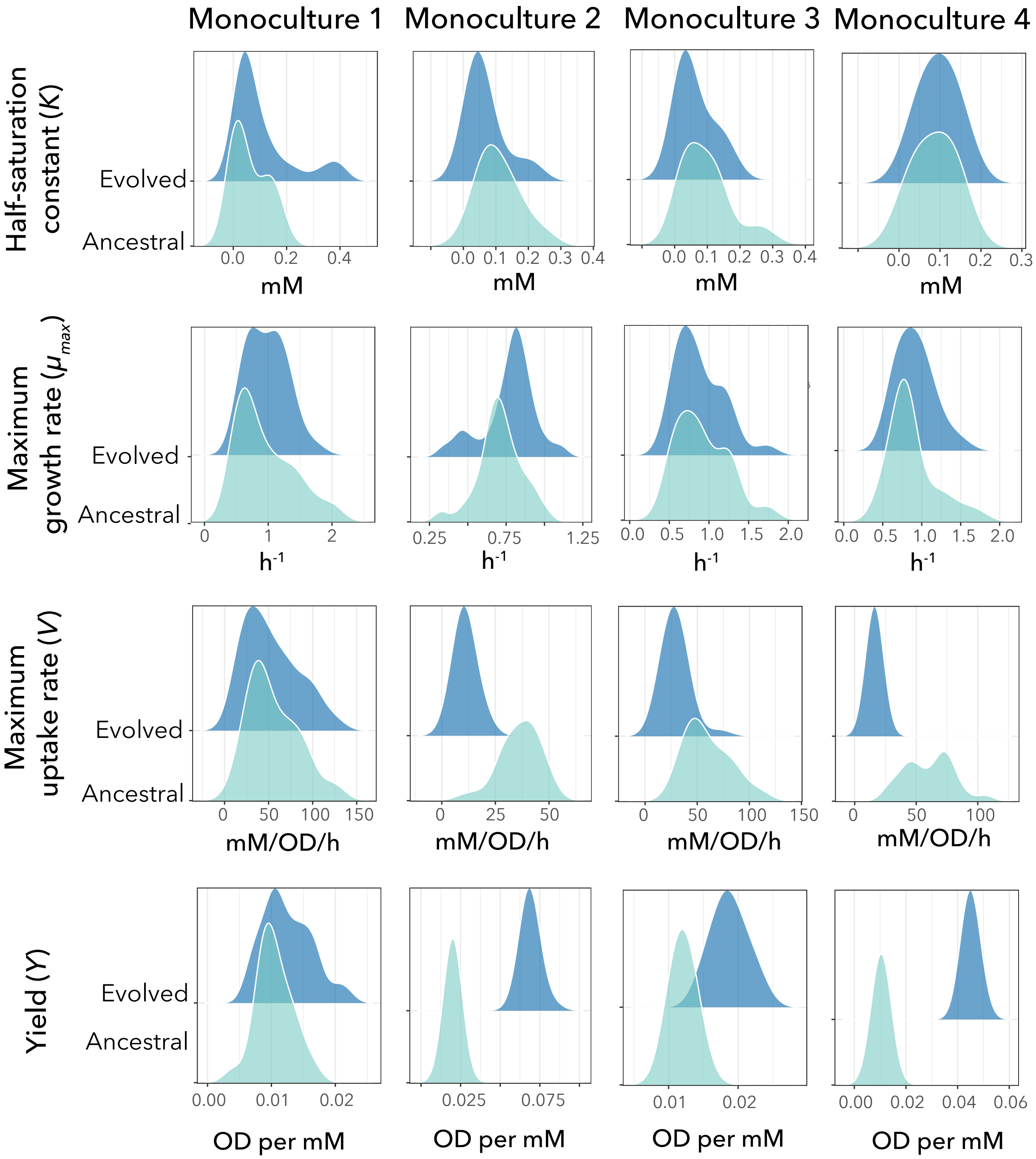

Fig. S3. Distributions of half-saturation constant, maximum growth rate, maximum uptake rate, and yield of the ancestral and evolved *P. putida* in monoculture. *P. putida* evolved a

higher yield in monoculture compared to its ancestor based on a t-test with *p*-values equal to 0.027, $2.2*10^{-16}$, $2.2*10^{-16,}$ and $2.2*10^{-16}$ for replicates 1, 2, 3, and 4, respectively. The distribution data were plotted using the `geom_density_ridges` from the `ggridges` package in R, which smooths the data.

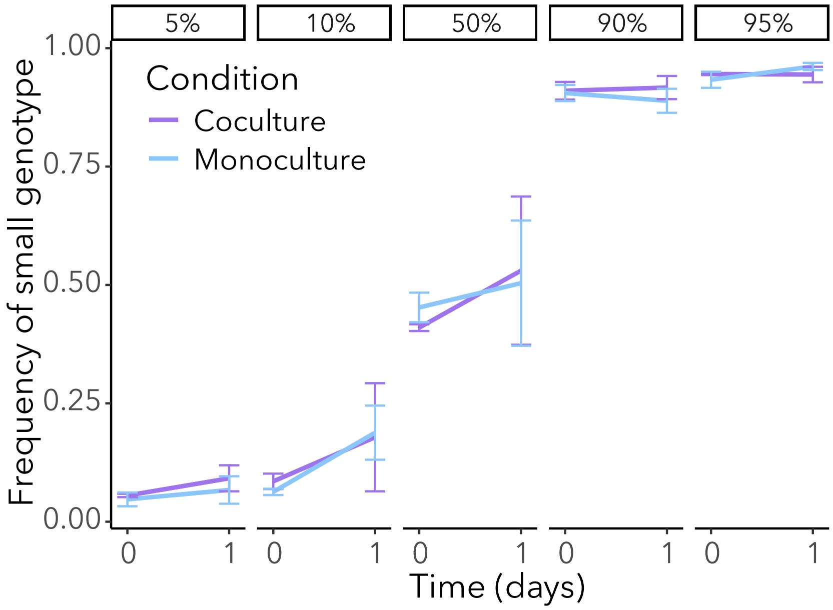

Fig. S4. Frequency of the small genotype in head-to-head competition against the *fleQ* genotype in monoculture and co-culture over one day. None of the genotypes can invade from rare either in monoculture or co-culture. The error bars show the standard errors of six replicate fractions (values between 0 and 1) of the small genotype.

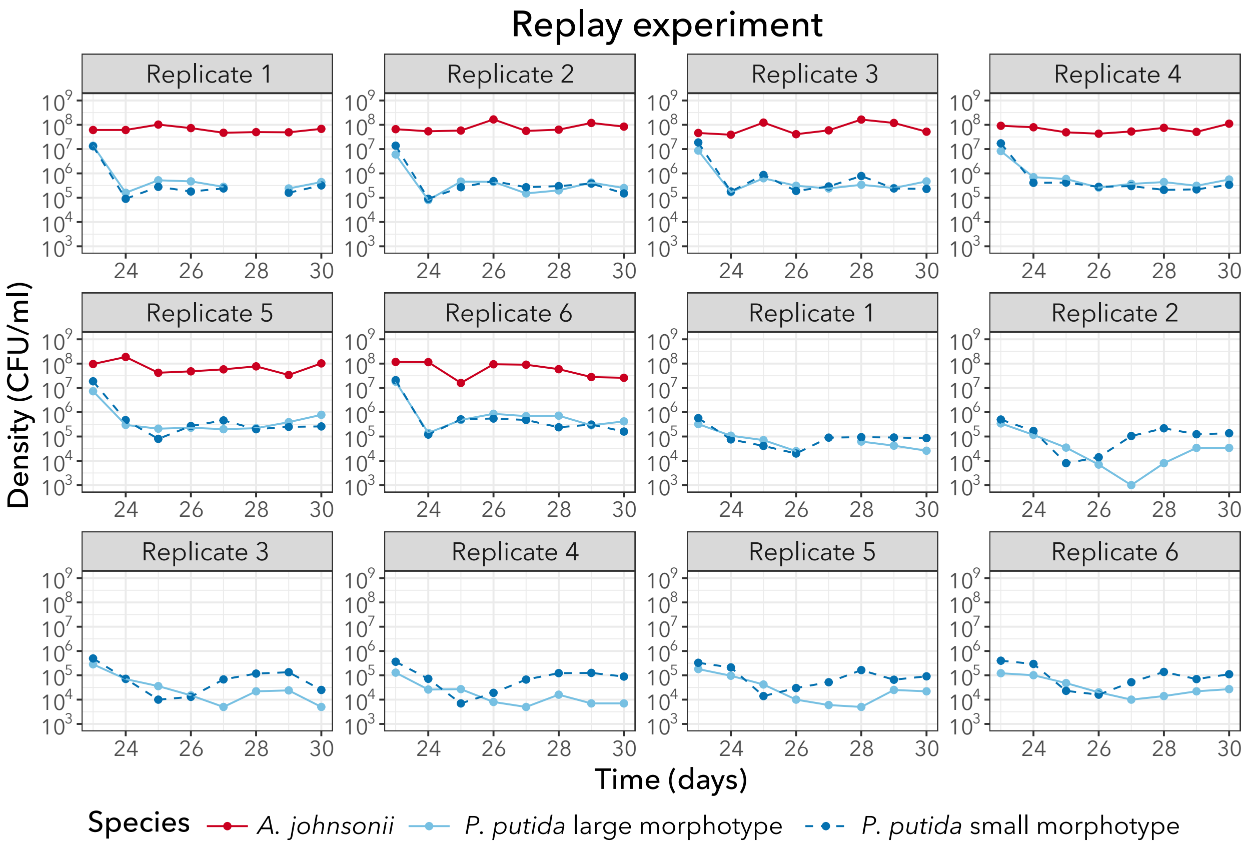

Fig. S5. Populations trajectories of six replicates with and without *A. johnsonii* over eight days (~ 50 generations). Each dot represents the final density (CFU mL^{-1}) of each type after daily cycles of growth.

Supplementary Tables

Table S1. Mutations identified in populations using *breseq*.

Position	Mutation	Freq.	gene	description
**Monoculture 1**				
1,640,523	M143R (ATG→AGG)	5.6%	*cmoB* ←	tRNA 5-methoxyuridine(34)/uridine 5-oxyacetic acid(34) synthase CmoB
1,640,524	M143L (ATG→CTG)	5.2%	*cmoB* ←	tRNA 5-methoxyuridine(34)/uridine 5-oxyacetic acid(34) synthase CmoB
2,768,267	G131G (GGT→GGG)	5.3%	*PP_RS12610* ←	TonB-dependent receptor
3,282,409	E169* (GAG→TAG)	10.4%	*PP_RS14970* ←	cupin domain-containing protein

3,990,242	+CCCGGCAG	39.0%	*PP_RS18280* →	helix-turn-helix domain-containing protein
4,647,573	Δ130 bp	5.5%	*[PP_RS21375]*	SulP family inorganic anion transporter
6,098,739	A117V (GCG→GTG)	11.9%	*hexR* ←	transcriptional regulator HexR
**Monoculture 2**				
139773	Q244P (CAG→CCG)	5.8%	*PP_RS00675* ←	ATP-binding protein
1,615,699	A18G (GCT→GGT)	7.3%	*PP_RS07310* ←	AbrB family transcriptional regulator
4,964,741	2 bp→C	100%	*fleQ* ←	transcriptional regulator FleQ
5,715,528	N65N (AAC→AAT)	100%	*tatB* →	Sec-independent protein translocase protein TatB
6,029,059	A134A (GCC→GCA)	100%	*PP_RS27510* ←	aldehyde dehydrogenase
**Monoculture 3**				
1,211,148	L440R (CTG→CGG)	5.5%	*PP_RS05530* ←	FMN-binding glutamate synthase family protein
1,640,517	I145S (ATT→AGT)	5.5%	*cmoB* ←	tRNA 5-methoxyuridine(34)/uridine 5-oxyacetic acid(34) synthase CmoB
1,640,524	M143L (ATG→CTG)	8.0%	*cmoB* ←	tRNA 5-methoxyuridine(34)/uridine 5-oxyacetic acid(34) synthase CmoB
3,823,579	L204R (CTG→CGG)	5.8%	*PP_RS17595* ←	sugar kinase
**Monoculture 4**				
1,843,830	D322H (GAC→CAC)	100%	*PP_RS08500* ←	sensor protein GacS
4,978,562:1	+C	100%	*flgH* ←	flagellar basal body L-ring protein FlgH
**Co-culture 1**				
1,663,184	L403V (TTG→GTG)	7.4%	*PP_RS07525* ←	MHS family MFS transporter
1,663,192	M400R (ATG→AGG)	8.3%	*PP_RS07525* ←	MHS family MFS transporter
3,282,468	Δ30 bp	26.9%	*PP_RS14970* ←	cupin domain-containing protein
3,282,843	R24H (CGC→CAC)	6.00%	*PP_RS14970* ←	cupin domain-containing protein
3,282,883	R11G (CGT→GGT)	15.6%	*PP_RS14970* ←	cupin domain-containing protein
4,964,681	A226V (GCC→GTC)	23.1%	*fleQ* ←	transcriptional regulator FleQ
**Co-culture 2**				
563,729	A271V (GCG→GTG)	20.8%	*rpoA* →	DNA-directed RNA polymerase subunit alpha
3,270,038	F19V (TTC→GTC)	5.4%	*PP_RS14890* ←	cupin domain-containing protein
4,635,466	G97D (GGC→GAC)	16.9%	*uvrY* ←	UvrY/SirA/GacA family response regulator transcription factor
4,964,300	L353Q (CTG→CAG)	35.6%	*fleQ* ←	transcriptional regulator FleQ

**Co-culture 3**				
1,843,368	G476C (GGT→TGT)	5.1%	*PP_RS08500* ← *(gacS)*	sensor protein GacS
1,844,178	Δ1 bp	5.2%	*PP_RS08500* ← *(gacS)*	response regulator GacS
2,768,251	F137V (TTC→GTC)	5.3%	*PP_RS12610* ←	TonB-dependent receptor
3,987,689	Δ1 bp	12.9%	*PP_RS18275* → *(olpA)*	hydantoinase/oxoprolinase family protein
4,635,160	T199I (ACC→ATC)	6.3%	*uvrY* ←	UvrY/SirA/GacA family response regulator transcription factor
4,635,644	S38P (TCG→CCG)	7.7%	*uvrY* ←	UvrY/SirA/GacA family response regulator transcription factor
**Co-culture 4**				
1,640,524	M143L (ATG→CTG)	5.7%	*cmoB* ←	tRNA 5-methoxyuridine(34)/uridine 5-oxyacetic acid(34) synthase CmoB
4,964,290	H356Q (CAC→CAG)	7.5%	*fleQ* ←	transcriptional regulator FleQ

Table S2. Average relative growth rate of the small genotype, *i*, in head-to-head competition with the *fleQ* genotype, *j*, in monoculture or co-culture. The values ± standard errors were obtained from three replicates.

Starting frequency	Condition	Selection rate constant r_{ij} (day^{-1})	p-value*
5%	Monoculture	0.30 ± 0.24	0.328
	Co-culture	0.45 ± 0.28	0.251
10%	Monoculture	1.12 ± 0.57	0.190
	Co-culture	0.43 ± 0.96	0.700
50%	Monoculture	0.18 ± 0.60	0.788
	Co-culture	0.52 ± 0.73	0.553
90%	Monoculture	-0.17 ± 0.46	0.750
	Co-culture	0.16 ± 0.19	0.489
95%	Monoculture	0.54 ± 0.52	0.402
	Co-culture	0.05 ± 0.25	0.848

* Two-sided t-test testing whether r_{ij} is different than zero.
